# Long noncoding RNA RP11‐547D24.1 regulates proliferation and migration in papillary thyroid carcinoma: Identification and validation of a novel long noncoding RNA through integrated analysis of TCGA database

**DOI:** 10.1002/cam4.2150

**Published:** 2019-05-01

**Authors:** Kai Guo, Lili Chen, Yunjun Wang, Kai Qian, Xiaoke Zheng, Wenyu Sun, Tuanqi Sun, Yi Wu, Zhuoying Wang

**Affiliations:** ^1^ Department of Head and Neck Surgery Fudan University Shanghai Cancer Center Shanghai China; ^2^ Department of Oncology Shanghai Medical College, Fudan University Shanghai China

**Keywords:** lncRNAs, papillary thyroid cancer, prognosis, RP11‐547D24.1

## Abstract

Long noncoding RNAs (lncRNAs) are known to be key regulators of numerous biological processes, and substantial evidence supports that abnormal lncRNA expression plays a significant role in tumorigenesis and tumor progression. However, the mechanism by which lncRNAs function in thyroid carcinoma are still unclear. To investigate the role of lncRNAs in the tumorigenesis of papillary thyroid carcinoma (PTC), we analyzed lncRNA data in The Cancer Genome Atlas RNA‐Seq database. A comparison of lncRNAs in cancerous thyroid tissues and normal tissues revealed hundreds of differentially expressed lncRNAs. Of 7589 lncRNAs identified in 561 thyroid cancer cases (503 cancerous tissues and 58 normal tissues), the expression levels of 144 were found to be aberrant (|log2 fold change| >2 and adjusted *P* < 0.05). The top 10 lncRNAs with the most significant differences were LINC01977, RP11‐363E7.4, RP3‐483K16.4, RP11‐547D24.1, RUNDC3A‐AS1, AC093609.1, CTD‐2008L17.2, HAGLROS, UNC5B‐AS1, and LINC01354. In addition, CTD‐2008L17.2, HAGLROS, AC093609.1, UNC5B‐AS1, and RUNDC3A‐AS1 were shown to play vital roles in determining the histological cancer type. Furthermore, RP11‐547D24.1 and UNC5B‐AS1 could distinguish patients with different stages of PTC. The lncRNA RP11‐547D24.1 was validated by loss‐of‐function assays, revealing that downregulation of this lncRNA regulates thyroid tumor cell proliferation and apoptosis, invasion, and migration. This study demonstrates the potential for using lncRNAs to interpret the pathogenesis and development of PTC.

## 1 INTRODUCTION

Thyroid cancer (TC), one of the most common endocrine carcinomas, stems from parafollicular or follicular thyroid cells, and its morbidity rate is increasing worldwide.^1^, ^2^ Papillary thyroid carcinoma (PTC) accounts for approximately 90% of all thyroid malignancies and has one of the most rapidly increasing incidence rates among all cancers.^4^ In most cases, the overall prognosis of patients with PTC is relatively good after surgical resection combined with radioactive iodine and levothyroxine treatment.^5^ Although treatment of PTC in early stages has achieved satisfactory outcomes, and patients with early‐stage PTC have a high overall survival rate, many patients are diagnosed at advanced stages.^6^, ^7^ In addition, 10%‐15% of PTC patients are still prone to relapse and distant metastasis, leading to a poor prognosis.^8^ Many studies have identified that genetic mutations and environmental factors are important in thyroid carcinogenesis, but many of the molecular mechanisms underlying TC pathogenesis remain unknown.^9^ Therefore, understanding the potential molecular mechanism underlying the oncogenesis and progression of TC is urgently needed.

Long noncoding RNAs (lncRNAs) are a type of RNA that exceed 200 nucleotides in length and cannot be translated into protein.^10^ While more than 70% of the human genome is reportedly transcribed into different types of RNA, less than 2% encodes protein.^11^, ^12^ LncRNAs have been validated in many human tissues and cells and may function in diverse, vital physiological and pathological processes, especially during tumor development.^13^, ^14^

Therefore, studying the functions and potential molecular mechanisms of lncRNAs can provide an important scientific basis for the clinical management of diseases, especially for tumors. Furthermore, high‐throughput RNA sequencing and microarray analyses of different types of cancerous tissues have revealed that thousands of lncRNAs are expressed aberrantly.^17^

To date, only a few lncRNAs in thyroid tumors have been identified. To discover more TC‐associated lncRNAs, we used The Cancer Genome Atlas (TCGA) RNA sequencing database of TC tissues and adjacent normal tissues to perform genome‐wide analyses and differentially profile lncRNA expression in TC.

We found the expression of RP11‐547D24.1 to be extremely upregulated in TC tissues compared with that in paratumorous (PT) tissues. Thus, we chose to use RP11‐547D24.1 in additional cell experiments. RP11‐547D24.1 is known to be located on human chromosome 1p36.33 and to have a 2318 bp transcript (hg38, Gencode Gene: ENSG00000233542.1). However, there have been no reports on the expression and biological function of the lncRNA RP11‐547D24.1 in PTC.

## 2 MATERIALS AND METHODS

### 2.1 Aberrantly expressed lncRNAs in PTC based on TCGA data

The high‐throughput RNA sequence data from confirmed PTC cases were downloaded from TCGA on 6 August 2017. These data were acquired on an Illumina HiSeq RNA‐Seq platform and included 502 PTC tissues and 58 adjacent noncancerous thyroid tissues. Currently, studies related to TCGA are widely acknowledged since TCGA is a community resource project.

PTC includes 60 483 mRNAs that cover 7589 lncRNAs, as described by RNA‐Seq data from the NCBI and Ensembl databases. We next assessed the differential expression of these lncRNAs with the R language package DESeq (adjusted *P* < 0.05 and absolute Log (2, FC)>2). Some lncRNAs were excluded from our analysis because their expression level fold changes were less than 1 in more than 10% of the samples. In addition, the expression level of each lncRNA was log 2 transformed for further analysis.

### 2.2 Clinical roles of the top 10 lncRNAs aberrantly expressed in PTC

To value the diagnostic effectiveness of the lncRNAs in PTC, we constructed receiver operating characteristic (ROC) curves and subjected the top 10 lncRNAs of the area under the ROC curve (AUC) to further analysis. Student's t test was used to analyze the top 10 lncRNAs differentially expressed between PTC and PT tissues. To further study the potential proteins related to RP11‐547D24.1, we downloaded genes from the CGC database (Cancer Gene Census, https://cancer.sanger.ac.uk/census) using the key word “thyroid”, and genes associated with the cancer pathway were also downloaded from the KEGG database (Kyoto Encyclopedia of Genes and Genomes, https://www.kegg.jp/kegg/). Pearson correlation analysis (STATA, version 12.0; Stata Corp, College Station, TX) was carried out to analyze the link between RP11‐547D24.1 and related mRNAs. For these lncRNAs, uni‐ and multivariate Cox analyses were also needed. The Kaplan–Meier method was utilized to reveal the prognostic significance of the lncRNAs, and the log‐rank test was conducted to analyze survival time.

### 2.3 Cell culture and transfection with RP11‐547D24.1 small interfering RNAs

The human cell lines NTHY, TPC‐1, and K1 were cultured in RPMI 1640 medium (Gibco; Thermo Fisher Scientific, Inc, Waltham, MA) supplemented with 10% fetal bovine serum (FBS; Gibco; Thermo Fisher Scientific, Inc), 10 units/mL penicillin and 10 mg/mL streptomycin at 37°C in a humidified 5% CO2 atmosphere. TPC‐1 and K1 cells were transfected with RP11‐547D24.1 siRNA (Shanghai, China) using Lipofectamine 3000 transfection reagent (Invitrogen; Thermo Fisher Scientific, Inc) in accordance with the manufacturer's protocol. All cell lines were plated in 6‐well plates before transfection. RP11‐547D24.1 cells were silenced by treatment with 10 μL of siRNA and 4 μL of Lipofectamine 3000 for 48 hours. The RP11‐547D24.1 siRNA sequences used in our research targeted the following sequences: forward 5′‐GAGUUAAAUAGAUAUCCAAdTdT‐3′ and reverse 5′‐UUGGAUAUCUAUUUAACU CdTdT‐3′.

### 2.4 Western blot assay

We used lysis buffer to extract protein, which was isolated with 10% sodium dodecyl sulfate polyacrylamide gel electrophoresis. The protein was then transferred to a polyvinylidene fluoride membrane, blocked with a primary anti‐active β‐catenin antibody (Millipore, Bedford, MA) overnight at 4°C and incubated with an anti‐mouse horseradish peroxidase‐conjugated secondary antibody (Cell Signaling Technology, Boston, MA) after being washed with Tris‐buffered saline at 37°C for 1 hour. Protein quantification was performed using an enhanced chemiluminescence reagent (Beckman Coulter, Brea, CA). GAPDH was used as a loading control.

### 2.5 Flow cytometry assay

The cells were washed with PBS and then fixed with Hank's buffered salt solution (137 mmol/L NaCl, 0.25 mmol/L Na2HPO4, 5.4 mmol/L KCl, 0.44 mmol/L KH2PO4, 1.0 mmol/L MgSO4, 1.3 mmol/L CaCl2 and 4.2 mmol/L NaHCO3) containing ice‐cold 80% ethanol for 30 minutes. The cells were then collected by centrifugation at 167.7×*g* for 10 minutes and washed with PBS 2 times. The cells were stained with propidium iodide (PI, 50 mg/mL; MultiScience Biotech, Hangzhou, China) containing 0.1 mg/mL RNase A and 0.6% NP‐40 (Thermo Fisher Scientific, Inc) in the dark for 30 minutes at room temperature, collected by centrifugation at 167.7×*g* for 10 minutes at 4°C, and washed with PBS. For the apoptosis assay, the cells were stained with FITC‐Annexin V and PI from the Annexin V‐FITC/PI kit (BD PharMingen, San Diego, CA). Flow cytometry was performed using a FACSCalibur flow cytometer (BD Biosciences, San Jose, CA). FlowJo 10.0.4 software (FlowJo LLC, Ashland, OR) was used to analyze the data. All procedures were performed 3 times.

### 2.6 Wound healing assay

TPC‐1 and K1 cells (1 × 10^6^ cells/well) were treated with the indicated reagents, and wounds were created using a 1 000 µL plastic pipette tip. The cells were then photographed every 12 hours from 0 hour to 36 hours. Five random fields of view were chosen, and the images were captured under microscopic magnification (×20). Experiments were carried out independently in triplicate.

### 2.7 Invasion assays

The ability of cells to invade was assayed by using Transwell chamber (Corning Costar Corp., Cambridge, MA) with coated Matrigel (BD Biosciences, Bedford, MA). The transfected cells were treated with trypsin/EDTA solution, washed once with serum‐free RPMI 1640 medium for centrifugation. A total of 1 × 105 cells were re‐suspended in 0.2 mL serum‐free RPMI 1640 medium and were seeded into the upper chamber. RPMI 1640 medium containing 10% FBS (0.5 mL) was added to the lower chamber as a chemoattractant. After 24 hours incubation at 37°C in a 5% CO2 incubator, cells on the top surface of upper chamber were removed by wiping with a cotton swab. Cells that invaded to the bottom surface of the filter were fixed with 4% paraformaldehyde for 10 minutes, stained in 0.1% crystal violet for 30 minutes, then washed in PBS and photographed by microscope. The values for invasion were obtained by counting 5 random fields per membrane. These experiments were performed 3 times in triplicate.

### 2.8 Tissue samples and RNA extraction, Quantitative real‐time PCR

Fifty PTC tissues and paired adjacent noncancerous thyroid tissue specimens were obtained from the Department of Head & Neck Surgery, Fudan University Shanghai Cancer Center. The paracancerous tissues were one cm from the edge of tumor, and there were no obvious cancer cells, as evaluated by an experienced pathologist. All tissue samples were snap‐frozen in liquid nitrogen immediately after thyroidectomy, and were transferred to the freezer at −80°C before use. All of the tissue specimens were obtained for this study with patient informed consent, and the use of the human specimens was approved by the Institutional Ethics Committee of Fudan University Shanghai Cancer Center and all procedures performed in our study were consistent with the ethical standards of our institutional research committee. Total RNA was extracted from PTC tissue and normal thyroid tissue with TRIzol reagent (Life Technologies, Carlsbad, CA), and the quality and concentration of RNA were assessed with a SmartSpec Plus spectrophotometer (Bio‐Rad, Hercules, CA). RNA purity was evaluated by the A260/A280 ratio. One microgram of total RNA was reverse transcribed using the All‐in‐One RNA RT‐quantitative real‐time PCR (qPCR) Detection Kit (GeneCopoeia Rockville, MD). qPCR was performed using a standard protocol from the SYBR Green PCR kit (Toyobo, Osaka, Japan) on Applied Biosystems 7300 real‐time PCR system (Applied Biosystems, Foster City, CA). β‐actin were used as references for mRNAs.

### 2.9 Statistical analysis

All data are representative of each assay repeated independently at least 3 times. Quantitative data are presented as the mean ± SD. We analyzed the data using STATA (version 12.0; Stata Corp, College Station, TX). Two‐tailed Student's t test was used to analyze the data between 2 groups. Categorical variables are expressed as frequency and percentage values. The chi‐square test or Fisher's exact test was used to describe the differences. *P* values < 0.05 were considered significant.

## 3 RESULTS

### 3.1 Aberrantly expressed lncRNAs in PTC based on TCGA data

**Figure 1:**
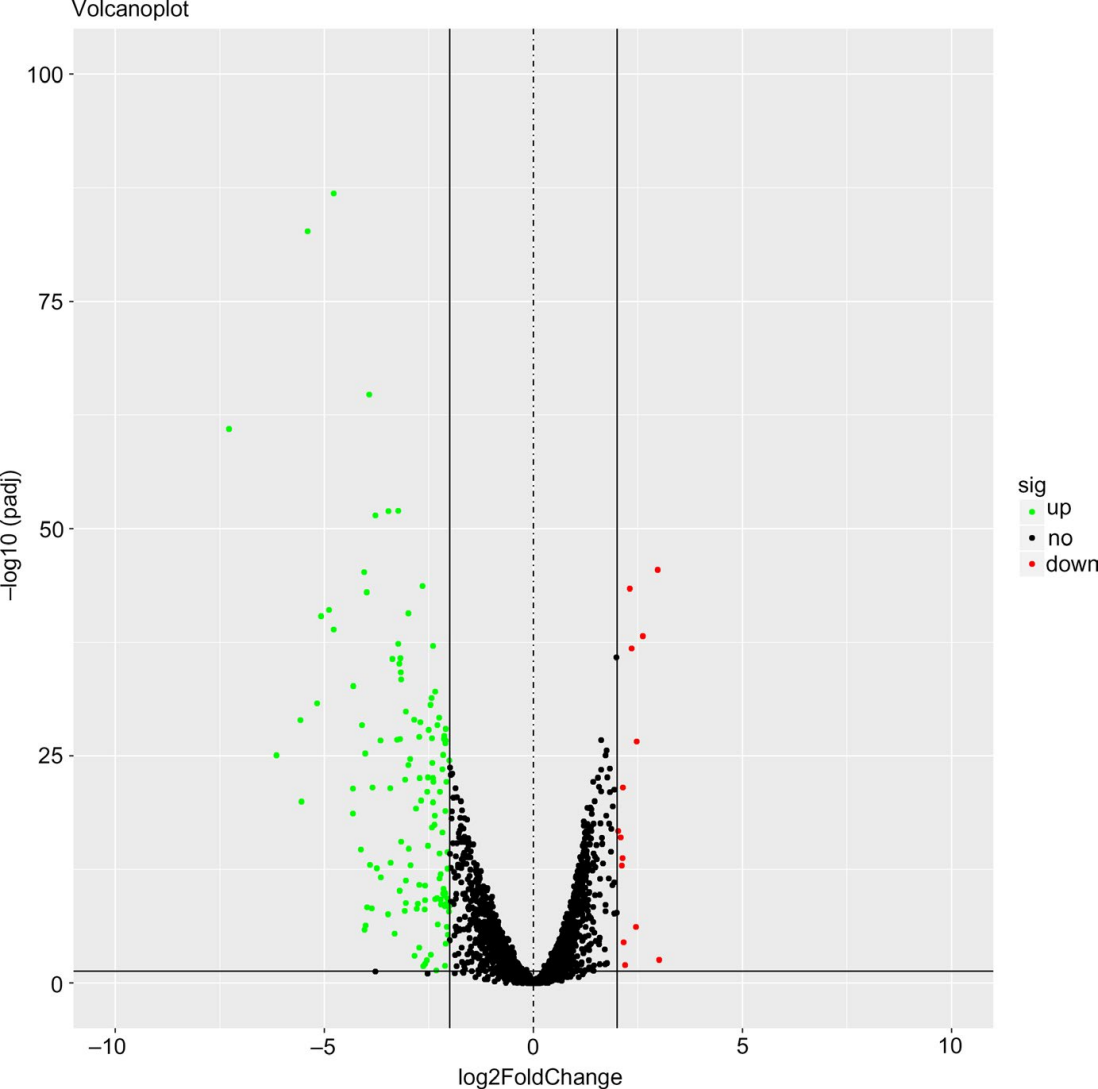
Volcano plot of the long noncoding RNAs (lncRNAs) aberrantly expressed between papillary thyroid carcinoma (PTC) and paratumorous thyroid tissues. Note: The green dots indicate high lncRNA expression, and the red dots indicate low lncRNA expression. The black dots show the lncRNAs with an expression of |log2FC|<2. The X axis represents an adjusted FDR, and the Y axis represents the log2FC value. Aberrantly expressed lncRNAs were identified by DESeq R. Altogether, 129 highly expressed and 14 low expressed lncRNAs were identified. This volcano plot was constructed using the ggplot2 package of R language

DESeq R was used to assess the expression level of each lncRNA. In total, 143 lncRNAs with aberrant expression (Figure 1) in PTC met the calculation condition, including 129 lncRNAs with high expression levels and 14 lncRNAs with low expression levels. ROC analysis was used to assess the lncRNAs with aberrant expression levels, and the top 41 lncRNAs are listed. LncRNAs with an AUC value greater than 0.90 (Table 1), which implied high diagnostic value, were selected.

**Table 1:** Analysis of 41 lncRNAs showing the most significant diagnostic value for PTC (AUC > 0.90)

Gene_name	AUC	Dysregulation	log2FC	*P* value	*P* adjusted
LINC01977	0.97	Up	3.93	1.15E‐68	1.87E‐65
RP11‐363E7.4	0.97	Up	2.01	3.75E‐27	3.15E‐25
RP3‐483K16.4	0.96	Down	−2.97	5.67E‐49	3.46E‐46
RP11‐547D24.1	0.96	Up	3.46	1.55E‐55	1.26E‐52
RUNDC3A‐AS1	0.96	Up	2.13	5.34E‐30	6.36E‐28
AC093609.1	0.96	Down	−2.31	9.37E‐47	4.15E‐44
CTD‐2008L17.2	0.95	Down	−2.62	2.35E‐41	6.74E‐39
HAGLROS	0.95	Up	3.85	4.78E‐24	2.99E‐22
UNC5B‐AS1	0.94	Up	5.17	1.12E‐33	1.82E‐31
LINC01354	0.94	Down	−2.02	4.87E‐19	1.86E‐17
AP000997.3	0.94	Up	3.99	2.62E‐46	1.06E‐43
AC018816.3	0.94	Up	3.19	1.30E‐29	1.40E‐27
AC079630.2	0.94	Up	5.40	7.66E‐87	1.87E‐83
LINC01539	0.94	Down	−2.47	2.83E‐29	2.76E‐27
LRP4‐AS1	0.94	Up	4.30	1.18E‐35	2.14E‐33
RP13‐895J2.3	0.93	Down	−2.14	4.67E‐24	2.96E‐22
RP11‐320N7.2	0.93	Up	3.37	1.08E‐38	2.30E‐36
RP11‐350J20.12	0.93	Up	4.78	4.32E‐42	1.32E‐39
CTC‐255N20.1	0.93	Up	3.23	1.19E‐55	1.16E‐52
RP11‐476D10.1	0.92	Up	6.14	9.69E‐28	8.59E‐26
CTC‐277H1.7	0.92	Up	2.29	3.50E‐31	4.49E‐29
RP11‐230G5.2	0.92	Up	5.57	8.44E‐32	1.18E‐29
AC068138.1	0.91	Down	−2.13	7.33E‐16	1.88E‐14
CTD‐3060P21.1	0.91	Up	2.44	2.74E‐34	4.61E‐32
AC079630.4	0.91	Up	4.04	1.10E‐48	5.97E‐46
RP11‐136I14.5	0.91	Up	2.85	7.90E‐32	1.13E‐29
LINC01770	0.91	Up	3.17	2.13E‐36	4.00E‐34
CTD‐2619J13.13	0.91	Up	4.10	3.26E‐31	4.29E‐29
RP11‐366L20.2	0.91	Up	2.99	5.90E‐44	2.06E‐41
RP11‐221N13.3	0.91	Up	4.78	2.96E‐91	1.44E‐87
CTD‐3014M21.1	0.90	Down	−2.35	6.33E‐40	1.54E‐37
AC007255.8	0.90	Up	5.08	1.33E‐43	4.34E‐41
RP11‐280O1.2	0.90	Up	7.28	8.66E‐65	1.06E‐61
RP11‐44N11.2	0.90	Up	4.31	6.93E‐24	4.17E‐22
RP11‐615I2.2	0.90	Up	2.40	3.14E‐40	8.05E‐38
AC008753.6	0.90	Down	−2.09	2.64E‐18	9.48E‐17
TYMSOS	0.90	Up	2.17	4.00E‐26	3.10E‐24
RP4‐751H13.7	0.90	Down	−2.16	6.12E‐06	3.31E‐05
RP11‐285E9.6	0.90	Up	2.71	1.57E‐31	2.13E‐29
DOCK9‐AS2	0.90	Up	2.10	2.03E‐29	2.02E‐27
TNRC6C‐AS1	0.90	Up	2.65	4.47E‐47	2.18E‐44

AUC, area under the ROC curve; lncRNAs, Long noncoding RNAs; PTC, papillary thyroid carcinoma.

### 3.2 Clinical value of the top 10 aberrantly expressed lncRNAs in PTC

**Figure 2:**
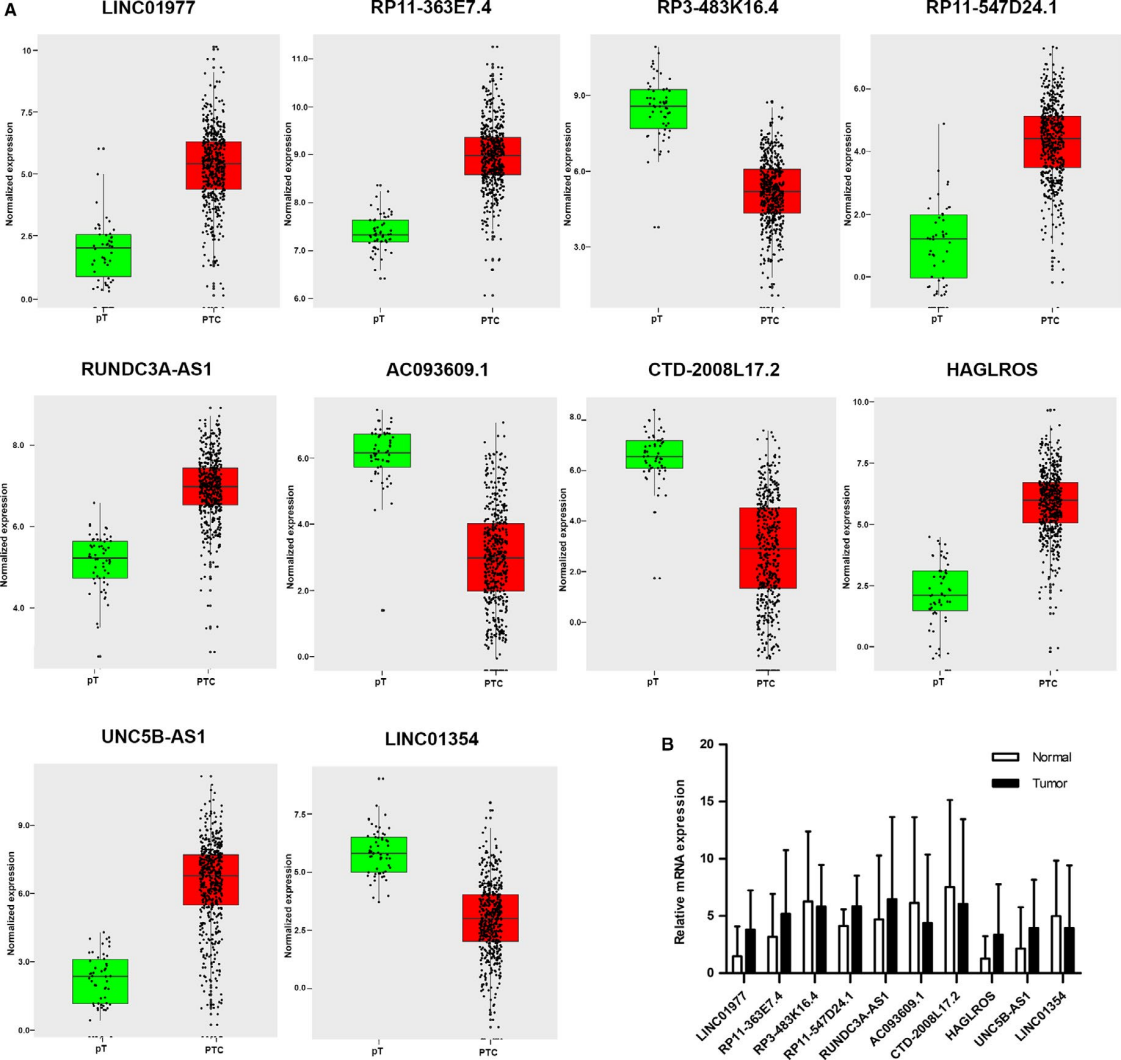
Different expression of the top 10 long noncoding RNAs (lncRNAs) between papillary thyroid carcinoma (PTC) and para‐tumorous PTC tissues. Note: A. Different expression of the lncRNAs in The Cancer Genome Atlas (TCGA) database. Red column indicates PTC tissues, and green column indicates para‐tumorous tissue (pT). The X axis indicates tissue types. The Y axis represents normalized expression of lncRNAs. This figure was drawn by ggplot2 package of R language. B. Clinical validation of the 10 lncRNAs in paired PTC and para‐tumorous PTC tissues. This figure was drawn by GraphPad Prism 5. *P < 0.05, **P < 0.01, ***P < 0.001

**Figure 3:**
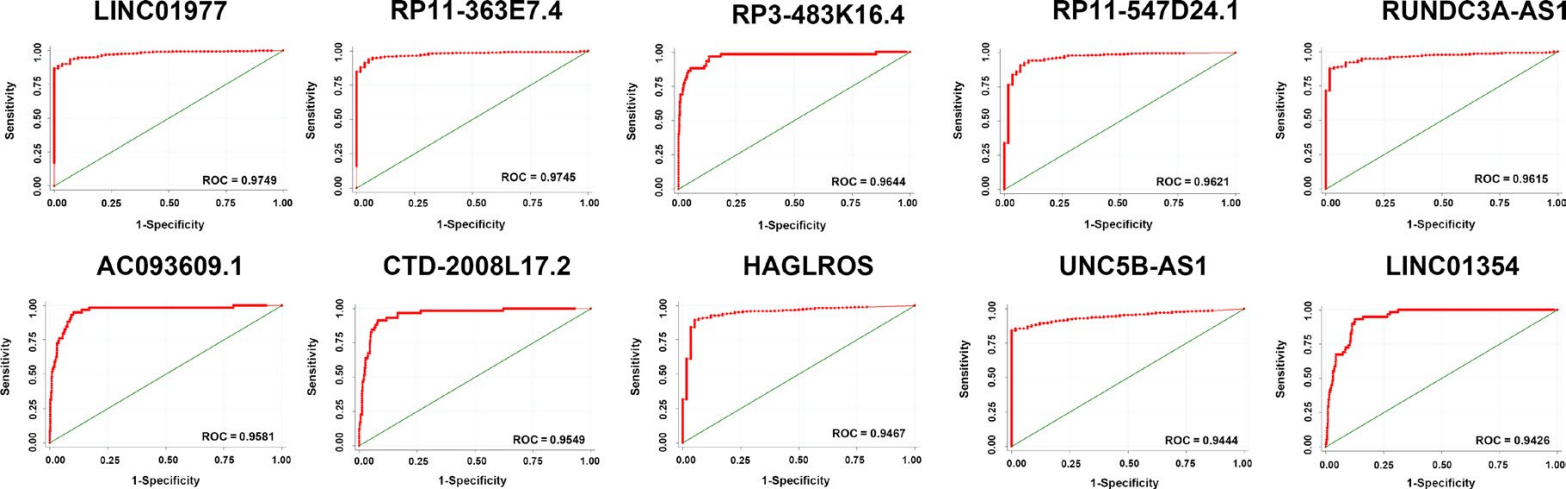
Receiver operating characteristic (ROC) curves of the top 10 long noncoding RNAs (lncRNAs) in papillary thyroid carcinoma (PTC) sorted by area under the ROC curve (AUC). Note: Red represents the sensitivity curve, and green indicates the identification line. The X axis shows the false positive rate, presented as “1‐Specificity”. The Y axis indicates the true positive rate, shown as “Sensitivity”. These curves were provided by STATA 12.0

**Figure 4:**
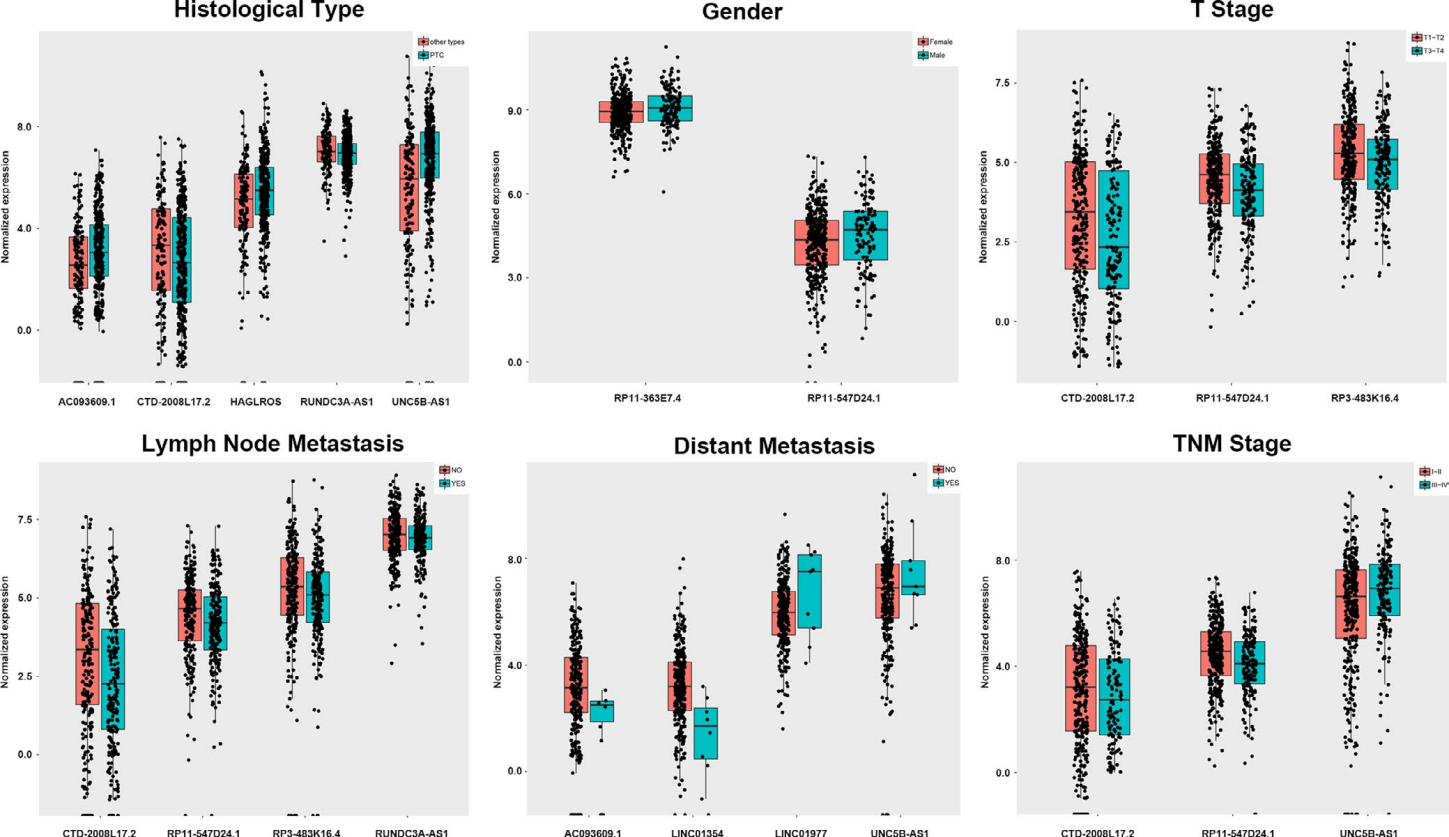
Association of the expression of key long noncoding RNAs (lncRNAs) with clinicopathological features of papillary thyroid carcinoma (PTC). Note: Statistically significant differences in several key lncRNAs were notably associated with various clinicopathological features: tumor stage (T1/T2 vs. T3/T4), lymph node metastasis (no vs. yes), pathological stage (I/II vs. III/IV), smoking status (no smoking vs. current smoking), and targeted molecular therapy (no vs. yes). The X axis indicates the different lncRNAs, and the Y axis indicates the normalized expression (log2). The plots were conducted using the ggplot2 package of R language. *P < 0.05, **P < 0.01, ***P < 0.001

The top 10 aberrantly expressed lncRNAs (Table 2), LINC01977, RP11‐363E7.4, RP3‐483K16.4, RP11‐547D24.1, RUNDC3A‐AS1, AC093609.1, CTD‐2008L17.2, HAGLROS, UNC5B‐AS1, and LINC01354, were selected for further analysis. The levels of LINC01977, RP11‐363E7.4, RP11‐547D24.1, RUNDC3A‐AS1, HAGLROS, and UNC5B‐AS1 were higher in PTC tissues than in PT thyroid tissues. In contrast, the levels of the other 4 lncRNAs were lower in PTC tissues than in PT thyroid tissues (Figure 2A). To verify the calculated data, 10 differentially expressed genes, were selected for q‐PCR in 50 pairs of PTC and adjacent noncancerous tissue samples. The 2^−ΔΔCt^ formula was used to calculate the differential gene expression. And Log_2_ (2^−ΔΔCt^) value was used for drawing the relative expression of ten selected genes in Figure 2B by GraphPad Prism 5. The results were consistent with the integrated data (Figure 2B). These 10 lncRNAs, which all had AUC values greater than 0.94 (Figure 3), showed potential for diagnostic use. Furthermore, multivariate Cox analysis indicated that LINC01354 could be an independent prognostic indicator of PTC (*P* = 0.037, Table 3). In addition, other lncRNAs may have a close relationship with some clinical parameters of PTC, such as gender, Histological type, TNM stage, Lymph node metastasis and Distant metastasis (Table 3, Figure 4). In particular, the levels of CTD‐2008L17.2 (*P* = 0.012), RP11‐547D24.1 (*P* = 0.000), and UNC5B‐AS1 (*P* = 0.031) could be used to distinguish PTC patients in early stages from those in advanced stages. CTD‐2008L17.2 (*P* = 0.007), RP11‐547D24.1 (*P* = 0.018), RUNDC3A‐AS1 (*P* = 0.029) and RP3‐483K16.4 (*P* = 0.012) were associated with lymph node metastasis. AC093609.1 (*P* = 0.040), LINC01354 (*P* = 0.013), UNC5B‐AS1 (*P* = 0.010), and LINC01977 (*P* = 0.028) had links to distant metastasis.

**Table 2:** Characteristics of the top 10 lncRNAs based on AUC size ranking

ENSEMBLE	Gene_name	AUC	Dysregulation	Fold change	*P* value
ENSG00000262772.1	LINC01977	0.97	Up	15.19	1.15E‐68
ENSG00000260912.1	RP11‐363E7.4	0.97	Up	4.03	3.75E‐27
ENSG00000271367.1	RP3‐483K16.4	0.96	Down	7.86	5.67E‐49
ENSG00000233542.1	RP11‐547D24.1	0.96	Up	11.02	1.55E‐55
ENSG00000267750.4	RUNDC3A‐AS1	0.96	Up	4.38	5.34E‐30
ENSG00000230587.1	AC093609.1	0.96	Down	4.96	9.37E‐47
ENSG00000206129.3	CTD‐2008L17.2	0.95	Down	6.15	2.35E‐41
ENSG00000226363.3	HAGLROS	0.95	Up	14.37	4.78E‐24
ENSG00000237512.5	UNC5B‐AS1	0.94	Up	36.06	1.12E‐33
ENSG00000231768.1	LINC01354	0.94	Down	4.07	4.87E‐19

AUC, area under the ROC curve; lncRNAs, Long noncoding RNAs.

**Table 3:** Relationship between the expression levels of the top 10 lncRNAs and clinicopathological features of PTC based on TCGA data

	Age		Gender		Histological type		Recurrence		Tumor size		Lymph node		TNM stage		Distant metastasis	
	*Z*	*P*	*Z*	*P*	*Z*	*P*	*Z*	*P*	*Z*	*P*	*Z*	*P*	*Z*	*P*	*Z*	*P*
CTD‐2008L172	0.24	0.813	1.23	0.220	2.10	0.036	−1.01	0.314	−2.84	0.005	−2.72	0.007	−2.51	0.012	−0.06	0.954
HAGLROS	1.96	0.050	1.38	0.166	−2.24	0.025	1.28	0.199	0.46	0.647	0.71	0.475	1.68	0.092	1.45	0.147
AC0936091	−0.50	0.616	0.35	0.723	−2.23	0.025	−1.81	0.070	−1.18	0.239	1.19	0.235	0.46	0.645	−2.06	0.040
LINC01354	0.08	0.933	−1.72	0.085	0.74	0.459	−2.08	0.037	−0.47	0.642	−0.81	0.415	−1.38	0.167	−2.48	0.013
RP11‐547D24.1	−0.52	0.600	2.67	0.008	1.90	0.058	−0.83	0.407	−2.91	0.004	−2.36	0.018	−3.57	0.000	−1.06	0.289
UNC5B‐AS1	0.64	0.524	1.13	0.258	−2.25	0.024	0.95	0.340	1.95	0.052	1.53	0.125	2.16	0.031	2.56	0.010
RP11‐363E74	−2.49	0.013	2.43	0.015	−1.72	0.086	1.30	0.192	0.40	0.686	0.08	0.933	−0.78	0.436	0.79	0.432
LINC01977	−1.87	0.062	1.70	0.088	−0.56	0.573	1.71	0.087	−0.12	0.906	0.71	0.480	0.07	0.941	2.19	0.028
RUNDC3A‐AS1	0.24	0.813	0.56	0.575	2.65	0.008	1.90	0.058	−0.58	0.560	−2.18	0.029	0.01	0.992	1.40	0.161
RP3‐483K164	−0.31	0.759	−0.08	0.937	1.23	0.22	−1.18	0.237	−3.09	0.002	−2.51	0.012	−1.92	0.055	0.30	0.765

lncRNAs, Long noncoding RNAs; PTC, papillary thyroid carcinoma; TCGA, The Cancer Genome Atlas.

### 3.3 WGCNA, gene ontology and KEGG pathway analyses of the aberrantly expressed lncRNAs and mRNAs in PTC

**Figure 5:**
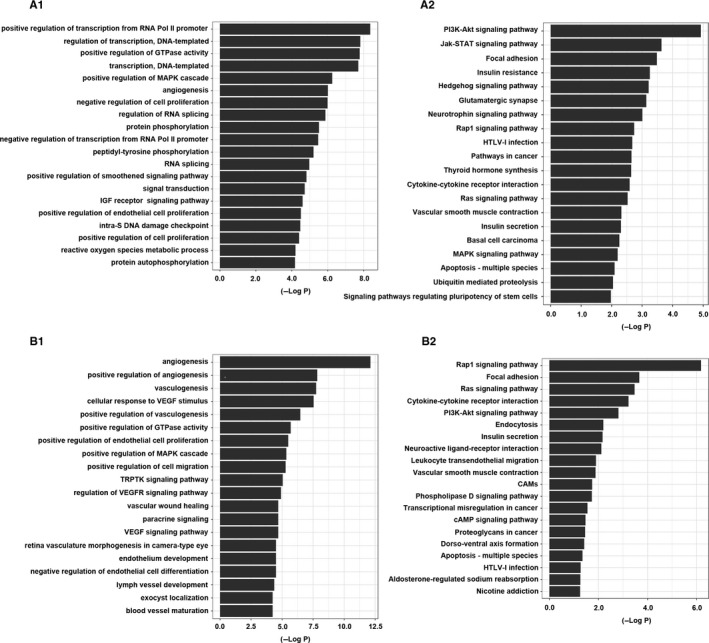
Go enrichment analysis (A) and KEGG pathway analysis (B) of all the mRNAs and RP11‐547D24.1 associated with papillary thyroid cancer. Note: Pol II: polymerase II; IGF: insulin‐like growth factor; TRPTK: transmembrane receptor protein tyrosine kinase; VEGF: vascular endothelial growth factor; VEGFR: vascular endothelial growth factor receptor; CAMs: cell adhesion molecules. A1: Significant gene ontology (GO) enrichment terms associated with all mRNAs. A2: Significant pathways associated with all mRNAs. B1: Significant GO enrichment terms associated with RP11‐547D24.1. B2: Significant pathways associated with RP11‐547D24.1

The genes coexpressed with the 10 lncRNAs were identified by weighted correlation network analysis (WGCNA), revealing 617 genes that are potentially coexpressed with these 10 lncRNAs in PTC. Among these genes, 37 had a relationship with AC0936091, and 255 had coexpression relationships with CTD‐2008L172 and with the other key lncRNAs (1 gene for HAGLROS, 200 genes for LINC01977, 2 genes for RP11‐363E7.4, 69 genes for RP11‐547D24.1, 21 genes for RP3‐483K16.4, 1 gene for RP3‐483K16.4, and 31 genes for UNC5B‐AS1). Biological annotation of the mRNAs identified from an integrated analysis of microarray data, especially for the lncRNA RP11‐547D24.1 in PTC, was performed using the DAVID online analysis tool; *P* < 0.05 was used as the cut‐off criterion. mRNAs were classified into 3 functional groups: molecular function, biological process, or cellular component. Significant results of the gene ontology (GO) enrichment and KEGG pathway analyses of mRNAs and lncRNAs in PTC are shown in Figure 5.

RP11‐547D24.1‐coexpressed genes were most enriched in the Rap1 signaling pathway, focal adhesion pathway and Ras signaling pathway. The most enriched GO terms for mRNAs coexpressed with RP11‐547D24.1 were angiogenesis, positive regulation of angiogenesis and vasculogenesis. Additionally, the most enriched GO terms were related to all of the top10 lncRNAs. Positive regulation of transcription by the RNA polymerase II promoter was the most enriched GO term.

### 3.4 Potential proteins related to RP11‐547D24.1

**Figure 6:**
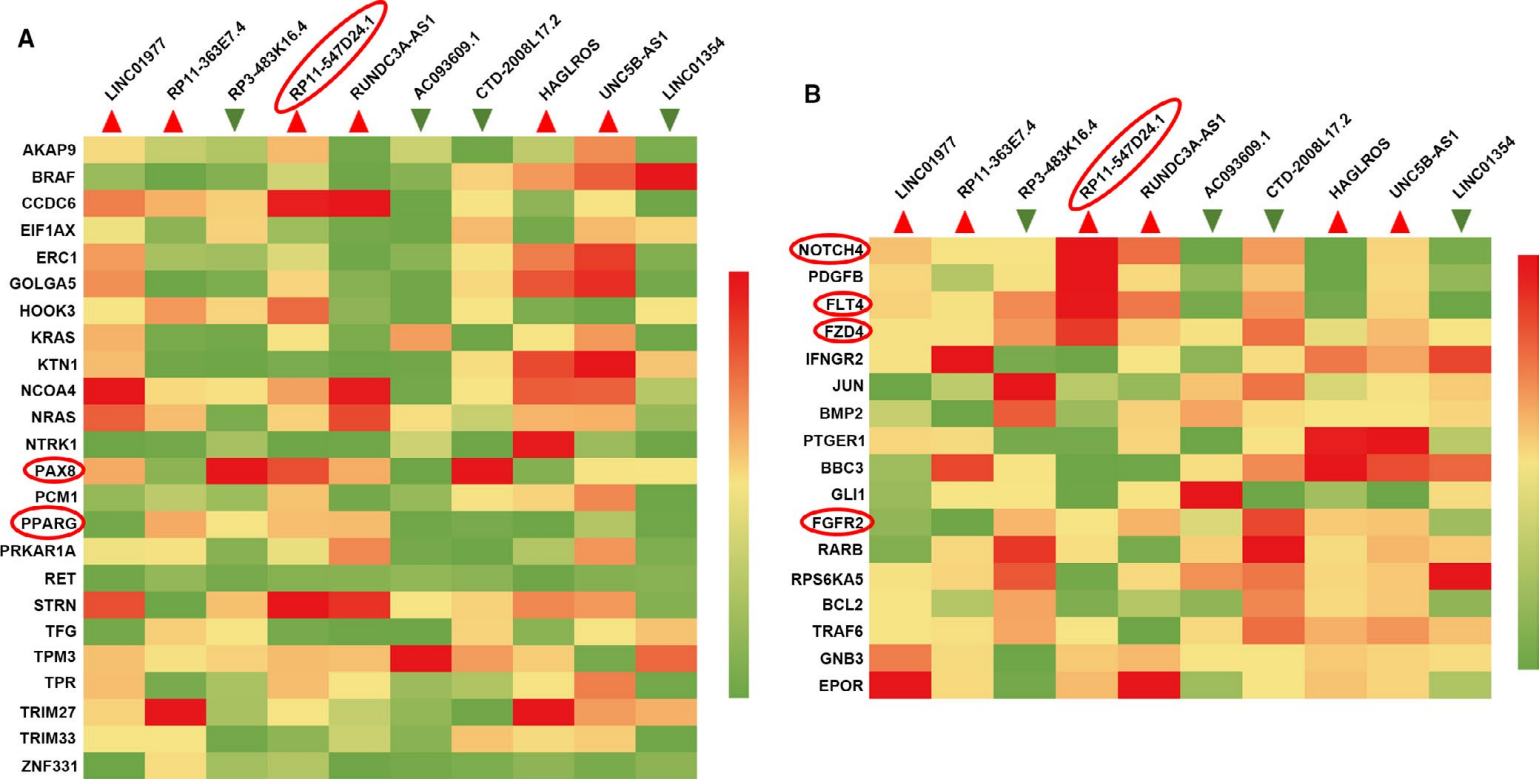
Heatmap showing long noncoding RNAs (lncRNAs) and signaling proteins associated with papillary thyroid carcinoma (PTC). Note: A. Relationships of proteins downloaded from the CGC database with the top 10 aberrantly expressed lncRNAs. B. Relationships of proteins downloaded from the KEGG database with the top 10 aberrantly expressed lncRNAs

**Figure 7:**
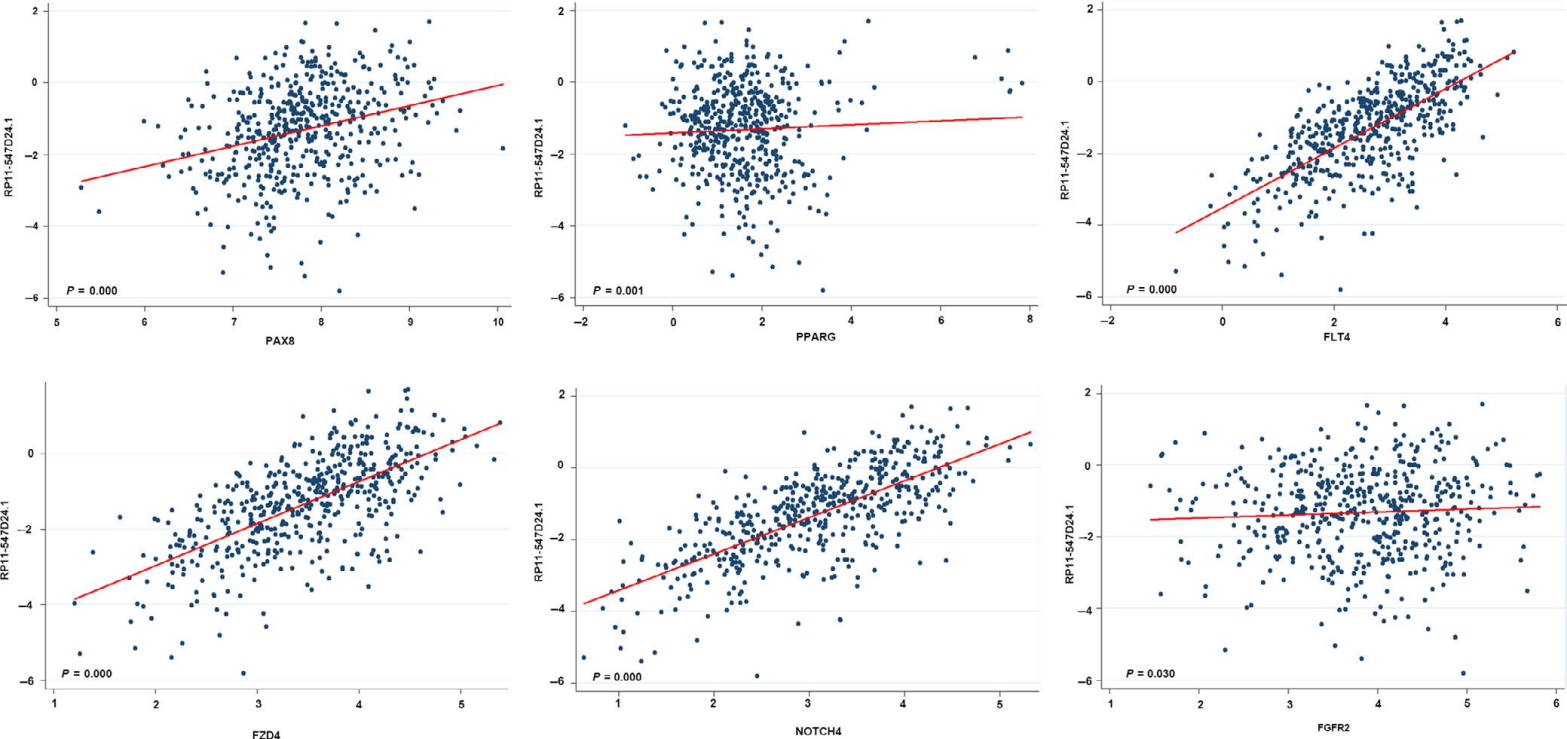
Correlation between RP11‐547D24.1 expression and PAX8/PPARG, FLF4, FGFR2, FZD4, and NOTCH4 in papillary thyroid carcinoma (PTC). Note: The expression of RP11‐547D24.1 was positively correlated with the expression of PAX8/PPARG, FLF4, FGFR2, FZD4, and NOTCH4 based on The Cancer Genome Atlas (TCGA) data

**Figure 8:**
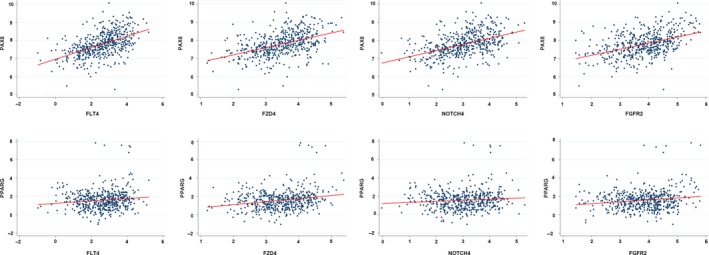
Correlation between PAX8/PPARG expression and FLF4, FGFR2, FZD4, and NOTCH4 in PTC

The CGC database is an ongoing effort to catalog genes that contain mutations causally implicated in cancer. We downloaded the CGC data from https://cancer.sanger.ac.uk/census and selected the genes with the key word “thyroid”. Furthermore, the genes associated with the cancer pathway were downloaded from the KEGG database (https://www.kegg.jp/kegg/). Then, the relationships of the 24 and 17 genes pulled from the CGC and KEGG databases, respectively, with the top 10 lncRNAs were calculated, and a heatmap was drawn using an illustrator (Figure 6). In the CGC data group, the expression of AKAP9, GOLGA5, HOOK3, KRAS, NCOA4, NRAS, PAX8, PCM1, PPARG, STRN, and TPM3 was apparently correlated with RP11‐547D24.1. Moreover, in the cancer pathway gene group, NOTCH4, PDGFB, FLT4, FZD4, FGFR2, RARB, TRAF6, GNB3, and EPOR were associated with the expression of RP11‐547D24.1. Based on the abovementioned results, we calculated the linear correlation between these proteins and RP11‐547D24.1 using TCGA data from the cBioportal database (http://www.cbioportal.org/). As shown in the figures, consistent results were found in PTC, revealing that the RP11‐547D24.1 level was markedly higher in PTC tissues than in PT tissues and positively associated with PAX8 (*P* = 0.000), PPARG (*P* = 0.001), FLT4 (*P* = 0.000), FZD4 (*P* = 0.000), NOTCH4 (*P* = 0.000) and FGFR2 (*P* = 0.03) (Figure 7). Furthermore, PAX8 and PPARG were associated with FLT4 (*P* = 0.000), FGFR2 (*P* = 0.000), FZD4 (*P* = 0.000) and NOTCH4 (*P* = 0.000) (Figure 8).

### 3.5 RP11‐547D24.1 regulates the cell cycle and cell proliferation

**Figure 9:**
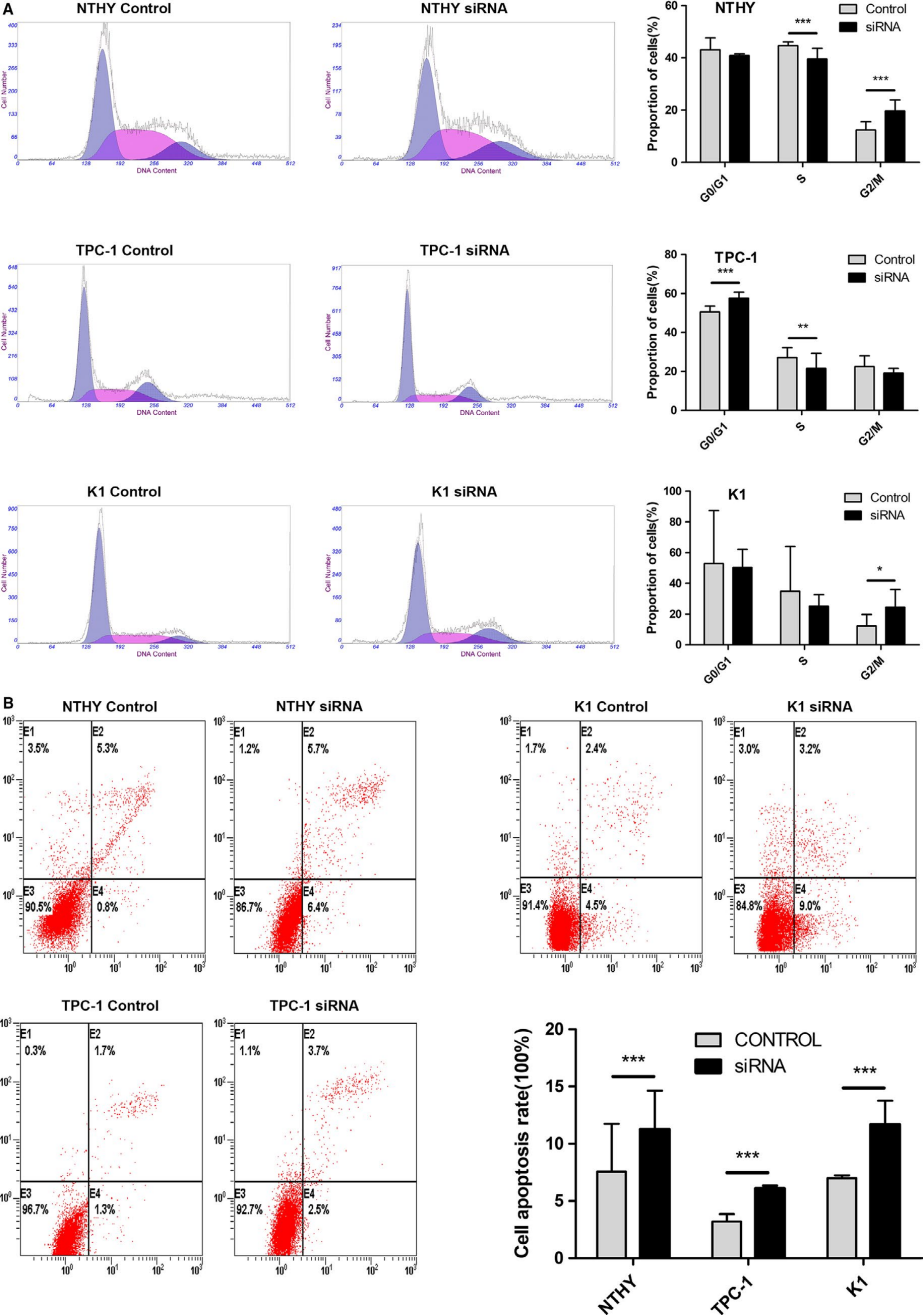
Effect of RP11‐547D24.1 on papillary thyroid carcinoma (PTC) cell cycle and apoptosis in vitro. Note: (A) Flow cytometry analysis showing the proportion of cells in the G0/G1, S and G2/M phases when RP11‐547D24.1 was silenced in NTHY, TPC‐1, and K1 cells. (B) For the cell apoptosis assay, NTHY, TPC‐1 and K1 cells were stained and analyzed by flow cytometry. E2, early apoptotic cells; E4, terminal apoptotic cells. The Y axis represents the apoptosis rate (E2 + E4)

**Figure 10:**
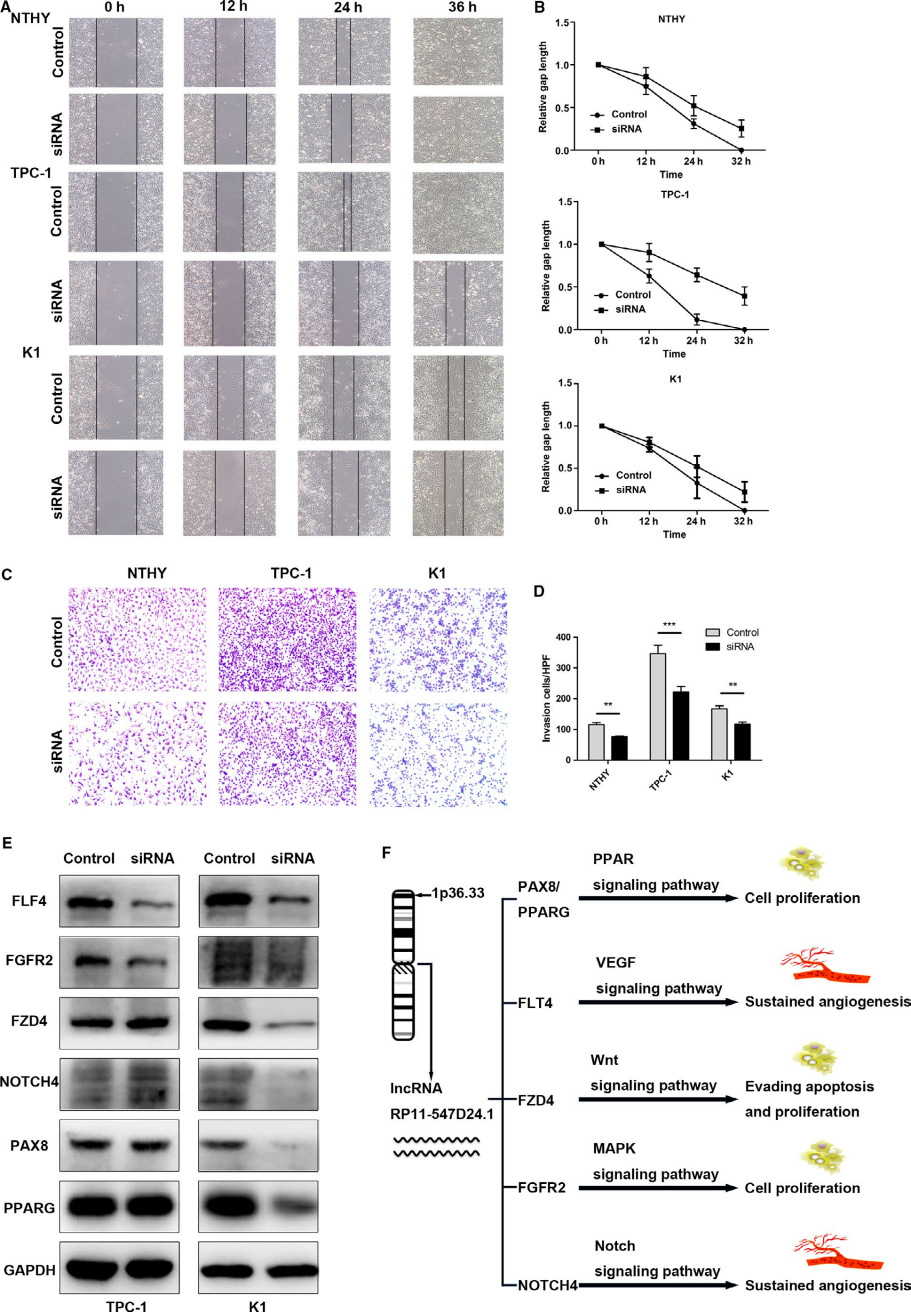
Effect of RP11‐547D24.1 knockdown on papillary thyroid carcinoma (PTC) migration and invasion; RP11‐547D24.1 negatively regulates the expression of tumor suppressor proteins (*eg*, PAX8/PPARG) in vitro. Note: (A and B). Cell migration was assessed using a wound‐healing assay. Images of the wounded monolayer were captured at 0, 12, 18, and 24 h after wounding for TPC‐1 cells and 0, 12, 24, and 36 h after wounding for K1 cells. The wound‐healing assay showed that RP11‐547D24.1 knockdown significantly suppressed PTC cell (TPC‐1 and K1) migration capacity. (C and D). Transwell migration assay measuring PTC cell migration in NTHY, TPC1, and K1 cells stably transfected with NC and siRNA, respectively. The number of migrated cells was evaluated by counting 10 random fields at ×100 magnification. (E). PAX8/PPARG, FLF4, FGFR2, FZD4, NOTCH4 expression levels in TPC‐1 and K1 cells were analyzed by western blot. (F). Hypothesis of pathway that RP11‐547D24.1 effect on PTC cell biological process. Values are shown as the mean (SD) from 3 independent experiments. *P < 0.05, **P < 0.01, ***P < 0.01

The frequent upregulation of RP11‐547D24.1 in PTC tissues suggests that RP11‐547D24.1 is significantly related to PTC tumorigenesis. Thus, the biological influences of RP11‐547D24.1 knockdown on regulating cancer cell were examined in vitro. Flow cytometry analysis indicated that depletion of RP11‐547D24.1 resulted in G0/G1 phase cells significant increases and S phase cells arrest in TPC‐1 cells (*P* < 0.05, Figure 9A). The proportion of apoptotic PTC cells was markedly increased by RP11‐547D24.1 knockdown (*P* < 0.05. Figure 9B), and the migration capacity was suppressed by silencing RP11‐547D24.1 in PTC cells (Figure 10A,B). The transwell invasion assay revealed that the invasiveness of PTC cells downexpressing RP11‐547D24.1 was significantly lower than that of the cells transfected with the empty control (*P* < 0.05, Figure 10C,D). These results indicated that RP11‐547D24.1 over‐expression significantly promoted the invasion of PTC cells in vitro. Immunoblot analysis showed that RP11‐547D24.1 knockdown reduced the levels of PAX8/PPARG, NOTCH4, FZD4, FGFR2 and FLF4 (Figure 10E). Accordingly, these data suggest that RP11‐547D24.1 promotes the growth and metastasis of PTC cells in vitro and may be related to the PPAR, VEGF, Wnt, MAPK, and Notch signaling pathways (Figure 10F).

## 4 DISCUSSION

To date, studies on sequencing the entire human genome have revealed that the noncoding elements of the genome are widely transcribed, yielding abundant lncRNAs.^18^ Mounting research indicates that dysregulation of lncRNAs contributes to a variety of biological activities, including tumorigenesis.^19^ Many lncRNAs are related to PTC, and their functions have been examined in recent studies. For example, lncRNA AB074169 could regulate cell proliferation by modulating KHSRP‐mediated p21 expression in thyroid cells and functions as a tumor suppressor during PTC tumorigenesis.^20^ The lncRNA NEAT1 promotes carcinogenesis and undesirable progression of PTC by modulating miR‐129‐5p/KLK7 expression.^21^ Furthermore, the highly expressed lncRNA AFAP1‐AS1 can promote the proliferation of tongue squamous carcinoma via regulating the Wnt/β‐catenin signaling pathway.^22^ NEAT1_2 is upregulated in PTC and positively related to the TNM stage and tumor size.^23^ The lncRNA HCP5 is overexpressed in PTC and promotes the proliferation, invasiveness, and angiogenic ability of PTC cells by functioning as a sponge for miR‐22‐3p, miR‐186‐5p, and miR‐216a‐5p and antagonizing their repression of ST6GAL2.^24^ Abnormal expression of the lncRNA BANCR has been demonstrated in colorectal, gastric and lung cancer, retinoblastoma, and PTC.^25^, ^26^ Elevated levels of BANCR were observed in human advanced malignant melanoma tissues, and melanoma cell proliferation and metastasis were demonstrated to be inhibited by the knockdown of BANCR via the mitogen‐activated protein kinase pathway.^27^

This study used a comprehensive analysis of lncRNA profiles in TC and identified abundant novel dysregulated lncRNAs, including the overexpressed lncRNAs LINC01977, RP11‐363E7.4, RP11‐547D24.1, RUNDC3A‐AS1, HAGLROS, and UNC5B‐AS1 and the downregulated lncRNAs RP3‐483K16.4, AC093609.1, CTD‐2008L17.2, and LINC01354. Furthermore, as some studies suggested that lncRNAs are potential predictive factors of survival, we demonstrated that the expression levels of the lncRNA LINC01354 may be closely related to TC recurrence.

To verify the analysis results, we investigated the function of the lncRNA RP11‐547D24.1 in PTC. The expression and function of RP11‐547D24.1 in PTC cell lines compared with that in controls were first examined in our study, revealing that RP11‐547D24.1 was significantly upregulated in both the TPC1 and K1 cell lines. In addition, silencing RP11‐547D24.1 significantly inhibited the proliferation, migration, and invasion abilities of PTC cell lines in vitro. The above results indicated that RP11‐547D24.1 act as a promoter of PTC progression. Furthermore, we showed for the first time that the function of RP11‐547D24.1 in PTC might be associated with the PAX8/PPARG, NOTCH, VEGF, and Wnt signaling pathways. Paired box 8 (PAX8) could affect the development of the kidney, eye, thyroid gland, central nervous system, and organs derived from the Müllerian duct.^28^ PAX8 could influence the expression of thyroid‐specific genes as a transcription factor.^29^, ^30^ Nonaka et al demonstrated that the expression levels of PAX8 in PTC, follicular thyroid carcinoma, and poorly differentiated thyroid carcinoma were invariable. While peroxisome proliferator‐activated receptor gamma plays a major role in the regulation of adipogenesis, its expression level in the normal thyroid is extremely low. The PAX8‐PPARg fusion protein is the product of a gene fusion between PAX8 and PPARG that regulates cell differentiation and lipid metabolism.^32^ In our study, knockdown of RP11‐547D24.1 could downregulate the expression of PAX8/PPARG, FLF4, FGFR2, FZD, and NOTCH4. All of these proteins are related to cancer pathways.

## 5 CONCLUSION

This study identified new mechanisms underlying TPC tumorigenesis. We found that the highly expressed lncRNA RP11‐547D24.1 could promote the development of malignant thyroid nodules from benign nodules by altering the proliferation of thyroid cells, which is potentially attributed to its ability to alter thyroid cell cycle progression. Targeted drugs for RP11‐547D24.1 can provide an important theoretical basis for clinical reversal of the malignant PTC phenotype.

## CONFLICT OF INTEREST

The authors have no conflict of interest to declare.
